# Advancing Immunotherapy in Chronic Lymphocytic Leukemia

**DOI:** 10.3390/ijms27093722

**Published:** 2026-04-22

**Authors:** Krzysztof Bieliński, Agnieszka Wysocka, Dawid Tyrna, Tadeusz Robak, Bartosz Puła

**Affiliations:** 1 Faculty of Medicine, Medical University of Warsaw, 02-091 Warsaw, Poland; krzysbielinski@wp.pl; 2 Department of General Hematology and Internal Medicine, Copernicus Memorial Hospital, 93-513 Lodz, Poland; ar.obracaj@kopernik.lodz.pl (A.W.); dm.tyrna@kopernik.lodz.pl (D.T.); robaktad@csk.umed.lodz.pl (T.R.); 3 Department of Hematology, Medical University of Lodz, Pabianicka 62 St., 93-513 Lodz, Poland

**Keywords:** chronic lymphocytic leukemia, immunotherapy, bispecific antibodies, monoclonal antibodies, chimeric antigen receptor T cells

## Abstract

The treatment of chronic lymphocytic leukemia (CLL) has significantly shifted from chemoimmunotherapy to targeted therapies like Bruton’s tyrosine kinase and BCL2 inhibitors. Despite these advancements, CLL remains an incurable disease characterized by immune dysregulation, therapeutic resistance, and cumulative toxicities. To overcome these challenges, novel immunotherapeutic strategies are emerging as fundamentally different approaches that target immune–tumor interactions. These innovations include novel monoclonal antibodies, bispecific antibodies that redirect T cell cytotoxicity, chimeric antigen receptor (CAR) T-cell therapies, and natural killer (NK) cell-based platforms. By actively engaging cellular cytotoxicity, these approaches show promise in high-risk and treatment-resistant scenarios where standard pathway inhibition is inadequate. Establishing optimal use, toxicity management, and combination strategies for these cell-engaging immunotherapies is now a critical priority in contemporary CLL research.

## 1. Introduction

Chronic lymphocytic leukemia (CLL) is the most prevalent leukemia in adults in Europe and North America and remains a biologically heterogeneous malignancy characterized by the progressive accumulation of mature, antigen-experienced B lymphocytes accompanied by profound immune dysregulation. Over the past decade, the therapeutic landscape of CLL has undergone a fundamental shift, moving from chemoimmunotherapy (CIT) to highly effective targeted agents, especially Bruton’s tyrosine kinase inhibitors (BTKis) and BCL2 inhibitors (BCL2is), which now form the core of international treatment recommendations. The clinical course of CLL is highly variable; a large number of patients, particularly those with mutated IGHV genes, experience an indolent trajectory, surviving for years without therapy, and some may never require it. Furthermore, those who progress to advanced states profit from currently used targeted treatments. Based on current clinical experience, BTKi and BCL2i are highly effective. Although they do not cure the disease, they provide most patients with significantly prolonged overall survival (OS) and a good quality of life [1,2]. Despite these advances, CLL remains incurable, and therapeutic resistance, treatment intolerance, and cumulative toxicity continue to be major factors driving disease progression and unmet clinical needs, prompting intense ongoing research by the scientific community [3,4,5,6,7].

Historically, immunotherapy using anti-CD20 monoclonal antibodies has played a crucial role in CLL management. Regimens such as fludarabine, cyclophosphamide, and rituximab (FCR), as well as bendamustine and rituximab (BR), have demonstrated durable responses in certain patient groups, especially in those lacking *TP53* pathway aberrations [8,9,10,11]. However, it has become increasingly clear that CIT-based approaches are associated with significant toxicity and lower efficacy in biologically high-risk diseases, especially in patients with *TP53* mutations or 17p deletion. These genetic abnormalities, now recognized as primary factors in treatment failure, confer resistance to DNA-damaging therapies and are strongly linked to aggressive disease biology and decreased survival [5,12,13,14].

The introduction of mechanism-driven targeted therapies has significantly improved outcomes for this high-risk group. Both BTKi- and BCL2i-based regimens have demonstrated substantial clinical effectiveness in *TP53*-aberrant CLL. These targeted approaches are now consistently recommended across major international guidelines, including those of iwCLL, ESMO, and NCCN, and local guidelines [3,4,5,6,15,16]. Recently, the combination of acalabrutinib and venetoclax (with or without obinutuzumab) has become a chemotherapy-free alternative to traditional regimens and other forms of targeted therapies utilizing other BTKis and BCL2is [17]. The recent breakthrough in the field is the development of BTK protein degraders. By inducing the proteasomal degradation of the entire BTK protein, these molecules effectively overcome resistance to both covalent and non-covalent BTKi. Crucially, specific novel degraders (such as NX-2127) also co-degrade immunomodulatory targets like Ikaros (IKZF1) and Aiolos (IKZF3), thereby actively stimulating T-cell activation alongside direct tumor cell killing [18,19].

Nevertheless, these therapies are not without limitations. Treatment-emergent toxicities, cardiovascular complications, infectious events, and acquired resistance mechanisms remain clinically significant, while double-refractory disease has become an increasingly challenging therapeutic scenario [17,18,20,21,22].

The latter problem can be partially addressed by using strategies focused on the tumor microenvironment (TME) [23]. Importantly, increasing evidence highlights the critical role of the TME in supporting leukemic cell survival and contributing to treatment resistance, suggesting that effective therapeutic strategies should also target microenvironmental interactions [24]. Simultaneously, immune dysregulation is a hallmark of CLL and seems to play a key role not only in disease persistence but also in accelerated CLL and the development of Richter transformation (RT) [23,25,26,27]. The latter presents a significant clinical challenge because of the aggressive clinical course associated with resistance to direct cytotoxic tumor-targeting therapies [28,29,30]. The interaction between malignant B cells and dysfunctional immune effector cells, especially T-cell exhaustion and impaired immune synapse formation, promotes immune escape and disease progression. In the context of RT, immune checkpoint inhibition targeting the PD-1/PD-L1 axis has emerged as a promising treatment option, with early clinical data indicating potential efficacy in this aggressive setting [31,32,33]. Collectively, these observations highlight the need for integrated treatment strategies that target both tumor-intrinsic mechanisms and the complex immunological and microenvironmental context of CLL. To date, no key prognostic factors for TME-oriented treatment approaches have been identified in CLL. As the number of double-exposed and double-refractory patients increases, novel TME-based treatment strategies are being developed.

These observations emphasize a key biological principle: CLL is not just a malignancy of B-cell proliferation but also a disorder of immune system balance. Significant dysfunction in both adaptive and innate immunity—including T cell exhaustion, impaired immune synapse formation, and dysregulated cytokine signaling—contributes to disease persistence, immune escape, and resistance to therapy [34,35,36,37,38,39]. Therefore, strategies that can restore or redirect effective antitumor immunity may be vital for overcoming resistance mechanisms and achieving long-lasting disease control [40,41]. In this context, immunotherapy becomes increasingly significant not just as an adjunct but as a fundamentally different approach targeting the immune–tumor interface.

**Figure 1:**
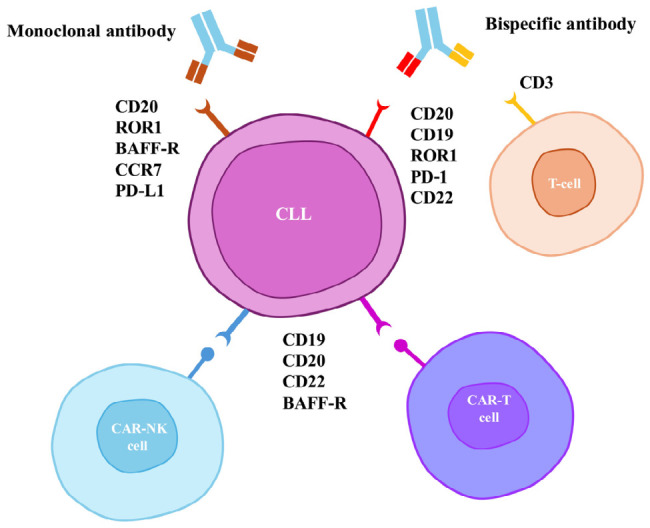
Therapeutic targets of immunotherapy in chronic lymphocytic leukemia. CLL—chronic lymphocytic leukemia; ROR1—receptor tyrosine kinase-like orphan receptor 1; CD—Cluster of Differentiation; BAFF-R—B-cell activating factor receptor; PD-1—programmed cell death protein 1; PD-L1—programmed death-ligand 1; CCR7—human chemokine receptor 7.

Recent advances have significantly broadened the immunotherapeutic options in CLL beyond traditional anti-CD20 antibodies. New monoclonal antibodies targeting disease-specific markers, bispecific antibodies (BsAbs) that activate T cell cytotoxicity, chimeric antigen receptor (CAR) T-cell therapies, and emerging natural killer (NK) cell platforms collectively constitute a new wave of immune-based strategies [42,43,44,45]. These approaches show particular promise in high-risk and treatment-resistant diseases, where pathway inhibition alone might be insufficient. However, their optimal integration into current treatment protocols remains to be determined and requires careful evaluation of efficacy, safety, and sequencing. Because CLL predominantly affects an older, often frail patient population, it is important to acknowledge the risks of possibly higher toxicities of CAR T-cell therapy in this demographic group [46]. Consequently, these intensive cellular therapies will likely be reserved for a minority of relatively young or fit patients presenting with aggressive or refractory disease [47]. In contrast, emerging immunotherapies with more favorable safety profiles have a much higher likelihood of entering standard care for the broader CLL population, enabling disease control while maintaining an acceptable long-term safety profile. In particular, novel monoclonal antibodies targeting antigens such as ROR1 and BAFF-R represent highly promising additions (Figure 1) [48,49,50].

In this review, we discuss the evolving role of immunotherapy in CLL, emphasizing its biological basis, clinical evidence, and future treatment strategies, with particular focus on how to address high-risk disease biology, resistance, and immune escape.

## 2. Novel Monoclonal Antibodies

The introduction of anti-CD20 monoclonal antibodies marked the first breakthrough in CLL immunotherapy. Monoclonal antibodies have transformed the treatment of CLL and are now used as monotherapy or in combination with other agents [51]. Development of innovative antibodies targeting new molecular markers is ongoing (Table 1).

### 2.1. Belimumab

B-cell activating factor (BAFF) is an essential cytokine in the tumor necrosis factor (TNF) family. Protective stromal cells and nurse-like cells within the lymph nodes and bone marrow secrete abnormally high levels of soluble BAFF [52]. When BAFF binds to its specific receptors on the surface of CLL cells—mainly the B-cell activating factor receptor (BAFF-R), but also transmembrane activator, calcium modulator and cyclophilin ligand-interactor (TACI), and B-cell maturation antigen (BCMA)—it activates the non-canonical NF-κB signaling pathway. This cascade increases the production of anti-apoptotic proteins, effectively protecting the leukemic cells from spontaneous apoptosis [53].

Belimumab is a human monoclonal antibody that targets soluble BAFF. By neutralizing circulating BAFF, a B-cell survival factor, belimumab prevents BAFF from binding to its receptors on lymphocytes. The drug is approved for the treatment of systemic lupus erythematosus (SLE). Preclinical studies, including those by Tandler et al., have shown that BAFF impairs the therapeutic efficacy of small-molecule inhibitors such as idelalisib, ibrutinib, and venetoclax, as well as monoclonal antibodies including rituximab, in patients with CLL. These results indicate that blocking BAFF with belimumab may restore or improve CLL cells’ sensitivity to these agents [54]. An ongoing phase II clinical trial is assessing the effectiveness of adding belimumab to a rituximab/venetoclax regimen in patients with relapsed or refractory CLL, with minimal residual disease (MRD) negativity as the primary endpoint (#NCT05069051). The estimated primary completion date is July 2026 [54].

### 2.2. Ianalumab 

Ianalumab is a fully human monoclonal antibody that targets BAFF-R and is extensively studied for autoimmune diseases, immune thrombocytopenia, and CLL. Its mechanism includes B-cell depletion, antibody-dependent cellular cytotoxicity (ADCC), interruption of BAFF-R-mediated signaling, and activation of NK and T cells. In a phase Ib trial, 39 patients with CLL who had not achieved a complete response (CR) or had developed resistance mutations after at least 1 year of ibrutinib therapy were enrolled. Patients received intravenous ianalumab combined with ibrutinib for up to six 28-day cycles. After reaching CR at 6 cycles, the drug was discontinued, and ibrutinib was continued for an additional 2 cycles. In patients who achieved undetectable minimal residual disease (uMRD) at the start of cycle 9, discontinuing ibrutinib was allowed at the investigator’s discretion. Among evaluable patients, the CR/CRi rate on day 1 of cycle 9 was 38%. uMRD in peripheral blood (PB) was achieved in 13 patients, with 8 patients achieving uMRD in both PB and bone marrow (BM). Of these, 6 patients were selected to stop ibrutinib and remained off therapy at the data cutoff [50].

### 2.3. CAP-100

CC chemokine receptor 7 (CCR7) and its ligands, CCL19 and CCL21, are essential for cell migration, entry, and sustained residency within lymph nodes [55]. The binding of these chemokines to CCR7 guides CLL cells into the TME, where they receive essential survival and proliferation signals, effectively shielding them from systemic therapies [56]. CCR7 expression in CLL is abnormally high relative to the corresponding normal CD5^+^ B-cell population or pan-B cells [57]. CAP-100 is a humanized IgG1 monoclonal antibody targeting the CCR7 receptor. Its primary mechanism of action is the competitive blockade of the CCR7 receptor, preventing the binding of the chemokines CCL19 and CCL21. In addition to neutralizing ligand-binding sites, CAP-100 activates ADCC [58]. In R/R CLL, ibrutinib may decrease CCR7 expression, while current data indicate that venetoclax treatment does not significantly change CCR7 expression patterns or CAP-100’s mechanism of action. This evidence indicates that CAP-100 could function as an adjunctive treatment to venetoclax, expanding the range of existing CLL therapies [59]. An ongoing phase Ia/Ib trial (#NCT04704323) aims to assess the safety and initial clinical benefits of CAP-100 monotherapy in patients with R/R disease who have received at least two prior standard systemic treatments. The results of this study have not yet been published.

### 2.4. Tislelizumab

Programmed cell death protein 1 (PD-1) is a crucial immune checkpoint receptor primarily found on activated T cells. When PD-1 binds to its ligands, PD-L1 or PD-L2, which are often expressed on tumor cells and within the TME, it sends inhibitory signals. This interaction effectively suppresses T-cell activity, enabling malignant cells to evade immune detection [60]. Blocking either PD-1 or PD-L1 inhibits this pathway, preventing the tumor from shutting down the immune response [61,62]. In the RT, malignant cells often express PD-1. Preclinical models strongly suggest that RT can be effectively targeted with immune checkpoint inhibitors [63]. Tislelizumab, a PD-1 monoclonal antibody approved to treat advanced esophageal squamous cell carcinoma, is also being investigated in hemato-oncology, particularly in combination with zanubrutinib for patients with RT. The combination of tislelizumab plus zanubrutinib was evaluated in an open-label, phase II study involving patients with RT who had received up to one prior RT-directed therapy. A total of 48 out of 59 patients who received at least two cycles of study treatment, including at least one dose in cycle three, were included in the analysis. Patients received tislelizumab intravenously at a fixed dose of 200 mg on day 1 of each 21-day cycle, in combination with zanubrutinib at a fixed dose of 160 mg twice daily from day 1 onward. The induction therapy consisted of six treatment cycles, followed by six consolidation cycles. The primary endpoint—overall response rate (ORR) at interim staging (after induction therapy)—was 58.3%, including 9 (18.8%) patients with CR and 19 (39.6%) with PR. The median progression-free survival (PFS) was 10 months, with a 12-month rate of 46.9%; the median OS was not reached (12-month OS rate, 74.7%). The most common side effects included hematological toxicities, gastrointestinal disorders, and infections [33].

### 2.5. Pembrolizumab

Pembrolizumab, an anti-PD-1 inhibitor, showed modest activity when combined with dinaciclib (a CDK2/5/9 inhibitor) in patients with R/R CLL during the phase Ib KEYNOTE-155 trial. Although the treatment was well tolerated, with any-grade adverse events (AEs) in 54 patients—mainly fatigue, nausea, and anemia—the ORR was 29.4%, and the median duration of response (DOR) was 10.3 months in R/R CLL. By the data cutoff, all patients had discontinued treatment, mostly due to progressive disease (PD) (52.8%). In conclusion, the ORR and DOR observed in the R/R CLL group suggest that pembrolizumab plus dinaciclib has limited antitumor activity [64,65].

### 2.6. Atezolizumab

Atezolizumab is a PD-L1 immune checkpoint inhibitor that was studied in a phase II trial involving treatment-naïve CLL patients (#NCT02846623). From July 2019 to February 2023, 37 patients were enrolled and received combination therapy with obinutuzumab, venetoclax, and atezolizumab. Of these, 31 patients completed 14 cycles of treatment, with 30 (97%) achieving BM uMRD. The median follow-up period was 40 months. The 4-year PFS and OS estimates were 89% and 94%, respectively. Atezolizumab was discontinued in 3 patients due to immune-related adverse events [66].

### 2.7. ABBV-319

The antibody–drug conjugate (ADC) ABBV-319 consists of a glucocorticoid receptor modulator (GRM) payload linked to a CD19 antibody. Its structure enables three distinct mechanisms of action that promote antitumor activity in B cells: delivering the GRM payload via CD19 to trigger apoptosis, inhibiting downstream CD19 signaling, and increasing ADCC through afucosylation of the antibody backbone. Unlike systemic glucocorticoids, ABBV-319 does not negatively impact NK cell function and may carry a lower risk of glucocorticoid-related adverse effects. Overall, these promising preclinical results support an ongoing phase I clinical trial (#NCT05512390) assessing the safety, tolerability, and early efficacy of ABBV-319 [67].

### 2.8. Zilovertamab

Zilovertamab (Zilo), also known as cirmtuzumab, is a humanized monoclonal antibody that targets receptor tyrosine kinase-like orphan receptor 1 (ROR1). ROR1, an oncofetal surface antigen, is absent in normal adult tissues but is abnormally expressed on various aggressive hematologic malignancies and solid tumor cells. Zilo, in combination with ibrutinib, was evaluated in a phase I/II clinical trial (#NCT03088878). Among patients with CLL, the ORR was 91.2%, and at a median follow-up of 31.4 months, the median PFS was not reached. In part 3 of the study, CLL patients were randomized 2:1 to receive Zilo plus ibrutinib (Zilo+Ibr) or ibrutinib alone (Ibr). At a median follow-up of 21.1 months in Part 3, median PFS was not reached in either the Zilo+Ibr or Ibr group. The data from CLL patients compare favorably with outcomes from Ibrutinib monotherapy. Notably, among patients with *TP53* mutations treated with Zilo+Ibr, the 30-month landmark PFS was 100%, a highly encouraging finding that supports further investigation of this combination in this high-risk patient population [68].

In an ongoing single-center phase II trial (#NCT04501939), investigators are assessing the effectiveness of cirmtuzumab combined with venetoclax as consolidation therapy for patients with CLL/SLL who have been on venetoclax for at least 12 months and have detectable residual disease (>0.01% leukemia cells). The primary endpoint is the uMRD rate after 6 months of combination therapy. The study is expected to conclude in July 2026.

### 2.9. Zilovertamab Vedotin

Zilovertamab vedotin (ZV) is an innovative ADC composed of zilovertamab, conjugated to the anti-microtubule cytotoxin monomethyl auristatin E (MMAE) via a protease-cleavable maleimidocaproyl-valine-citrulline-para-aminobenzoate linker—a highly potent anti-mitotic agent. This compound is currently being studied in several clinical trials involving patients with B-cell lymphomas. Results from the phase I WAVELINE-001 trial showed that ZV has a manageable safety profile and promising antitumor activity in heavily pretreated patients with diffuse large B-cell lymphoma (DLBCL), mantle cell lymphoma (MCL), and RT. A total of 56 patients were enrolled in Schedule 1; after 14 months of follow-up, the ORR was 29%, 53%, and 57% for DLBCL, MCL, and RT, respectively. Treatment-related AEs were reported in 41 patients (73%), with grade 3/4 AEs in 27 patients (48%), the most common of which were cytopenias. Although the results in the RT cohort are very promising, it should be noted that this group included only 7 patients [69]. In the ongoing phase II WAVELINE-006 trial, ZV is being evaluated either as monotherapy or in combination with nemtabrutinib in patients with R/R B-cell malignancies. Currently, only preliminary data from Cohort C, which enrolled 28 patients with R/R MCL, have been reported. The median time from the first dose to data cutoff was 7.3 months. In addition to expected hematologic toxicities, 19 patients (68%) developed peripheral neuropathy-related events, including symptoms such as gait disturbance, muscle weakness, peripheral neuropathy, paresthesia, peripheral sensory neuropathy, and polyneuropathy. Grade 3/4 neuropathic events occurred in 3 patients (11%). The ORR was 64%, with CR and PR observed in 9 patients (32% each) [70]. Data for patients with R/R CLL and follicular lymphoma (FL) (Cohort D) are currently maturing and have not yet been reported.

**Table 1:** Clinical trials evaluating the combination therapies using monoclonal antibodies in B-cell malignancies.

Agent	Phase	Antibody Target	No. of Participants	ORR	CR/CRi	PFS	OS	Most Common AEs [%]	Ref.
Ianalumab + ibrutinib	Ib	BAFF-R	39 (expansion n = 24)	N/A	37.5%	N/A	N/A	Gr. ≥ 3 TEAEs 41.0% TRAEs: 23.1%	[50]
Tislelizumab + zanubrutinib	II	PD-1	59 (enrolled); 48 (comprised in full analysis set)	58.3%	18.8%	Median: 10 months; 46.9% at 1 year	Median: not reached; 74.7% at 12 months	Gr. ≥ 1 TEAEs 98.2% (n = 57) Gastrointestinal disorders: 56.1% Infections: 78.9% with UTI 21.1% Pyrexia: 19.3% Peripheral edema: 17.5% Anemia: 19.3% Neutropenia: 21.1% Thrombocytopenia: 19.3%	[33]
Pembrolizumab + dinaciclib	Ib	PD-1	72 (enrolled); 17 (R/R CLL), 38 (R/R DLBCL), 17 (R/R MM)	29.4% (R/R CLL)	N/A	Median: 5.2 months (R/R CLL)	Median: 21.7 months (R/R CLL)	Gr. ≥ 3 TEAEs: 32% (n = 12); Lymphopenia: 13%; Neutropenia: 11%; Thrombocytopenia: 8%; Leukopenia: 8%; Laboratory TLS: 5%	[64,65]
Atezolizumab + obinutuzumab	II	PD-L1	37 (enrolled)	N/A	N/A	94% at 2 years; 89% at 4 years	94% at 2 years; 94% at 4 years	Gr. ≥ 3 TEAEs: Neutropenia: 59%; Thrombocytopenia: 32%	[66]
Zilovertamab + ibrutinib	I/II	ROR1	70 (enrolled), 34 (CLL), 26 (R/R MCL)	91.2%	8.8%	Median: not reached	N/A	Gr. ≥ 3 TEAEs Hypertension:10.6%; Pneumonia: 7.1%; Neutropenia: 5.9%; Atrial fibrillation: 5.9%; Fatigue: 5.9% TEAEs due to Zilo: 23.5% (CLL)	[68]
Zilovertamab vedotin + nemtabrutinib	I	ROR1	56 (enrolled); 17 (DLBCL); 17 (MCL); 7 (RT)	57% (RT)	14% (RT)	Median: 4.7 months (RT)	Median: 19.4 months (RT)	Gr. ≥ 3 TEAEs: 48% (n = 27); Neutropenia: 32%; Thrombocytopenia: 11%	[69]

ORR—overall response rate; CR/CRi—complete remission; PFS—progression-free survival; OS—overall survival; Ref.—reference; Gr.—grade; TEAEs—treatment-emergent adverse events; TRAEs—treatment-related adverse events; N/A—not available; CLL—chronic lymphocytic leukemia; DLBCL—diffuse large B-cell lymphoma; MM—multiple myeloma; RT—Richter transformation; MCL—mast cell leukemia; R/R—relapsed or refractory; ROR1—receptor tyrosine kinase-like orphan receptor 1; BAFF-R—B-cell activating factor receptor; PD-1—programmed cell death protein 1; PD-L1—programmed death-ligand 1.

## 3. Bispecific Antibodies

A relatively new class of agents that could potentially be highly effective in CLL therapy is T cell engagers (TCEs) [71]. In this group, BsAbs, already widely used in other hematological malignancies (especially in relapsed or refractory cases) such as multiple myeloma (talquetamab, teclistamab, elranatamab), follicular lymphoma (mosunetuzumab), and DLBCL (epcoritamab, glofitamab), are involved in several promising CLL clinical trials [72]. BsAbs (also called trispecific and tetraspecific antibodies) function by redirecting the physiological function of polyclonal cytotoxic T lymphocytes toward malignant B-cells via artificial connections between these two subgroups of white blood cells. The main advantage of BsAbs over CAR T therapies is the avoidance of ex vivo procedures—they are based on antibody structure, synthetically manufactured, and available “off-the-shelf”. Their components allow them to bind to multiple antigens and influence other metabolic pathways, which could be beneficial in the context of double-refractory CLL [73]. Numerous BsAbs are being explored as a potential CLL therapeutic option (Table 2).

### 3.1. Epcoritamab

Epcoritamab is a full-length CD20×CD3 IgG1 T-cell-engaging bispecific antibody. It binds simultaneously to CD20 on malignant cells and the CD3 subunit of the T-cell receptor (TCR) complex on endogenous T cells. This crosslinking activates T lymphocytes, resulting in the secretion of perforin and granzyme B, which induce apoptosis in the targeted leukemia cells [74].

To date, the strongest evidence for the efficacy and safety of BsAbs in CLL treatment comes from epcoritamab clinical trials. In the multicenter, open-label, phase Ib/II EPCORE CLL-1 trial (#NCT04623541), the medication was given to 42 patients with RT [75]. Thirty-two patients were male, with a median age of 69 years. The median time from disease onset to RT was 7.6 years. Epcoritamab was administered subcutaneously in a step-up dosing regimen. In 50% of cases, epcoritamab was used as a first-line treatment. At a median follow-up of 22.9 months, the ORR was 47.6%. In the first-line population and in subsequent lines of therapy, the ORRs were 57.1% and 38.1%, respectively. In patients with baseline *TP53* aberration and/or del(17p), the ORR reached 40%. The most common grade 3 or 4 AEs according to CTCAE were neutropenia (45%), anemia (38%), thrombocytopenia (38%), infection (21%), pneumonia (10%), and COVID-19 (5%). Cytokine release syndrome (CRS) was observed in 86% of patients, with only 7% experiencing grade 3. Immune effector cell-associated neurotoxicity syndrome (ICANS) of grade 1–2 occurred in 5 patients (12%), and only 2 developed clinical tumor lysis syndrome (TLS). Although three fatal AEs were reported, investigators stated that none were considered related to the study treatment.

In the EPCORE CLL-1 clinical trial, the effectiveness of epcoritamab combined with lenalidomide (Arm 2B) and epcoritamab combined with R-CHOP (Arm 2C) in patients with RT was examined [76]. Arm 2B involved 11 patients—6 male (55%) and 5 female (45%)—with a median age of 74 years. Prior therapy for CLL or SLL was given to 6 patients (55%). With a median follow-up of 12.6 months, the ORR was 82%, and the CR rate was 73%. The estimated median PFS was 5.7 months, while the median OS was not reached. Common AEs included CRS (100%, with grade 3/4 in 2 patients), neutropenia (82%), thrombocytopenia (73%), anemia, and hypokalemia (45% each). ICANS occurred in 2 patients (grade 1/2), and there was 1 fatal AE. Arm 2C involved 30 patients—22 male (73%) and 8 female (27%)—with a median age of 72 years. Prior therapy for CLL or SLL was administered to 17 patients (56%). With a median follow-up of 10.1 months, the ORR was 73%, and the CR rate was 60%. The estimated median PFS was 9.9 months, and median OS was 16.4 months. Common AEs included CRS (60%), anemia (60%), neutropenia (60%), diarrhea (33%), and febrile neutropenia (30%). ICANS occurred in 4 patients (grades 1/2/3), and fatal AEs occurred in 3 patients.

In the same clinical trial, epcoritamab was also given to 40 patients with R/R CLL [45]. The expansion cohort included 23 patients, while the optimization cohort had 17 patients. An additional cohort was created to reduce CRS by implementing a step-up dose. The median follow-up was 22.8 months for the expansion group and 2.9 months for the optimization group. The median age was 71.5 years. Before receiving epcoritamab, patients had a median of 4 lines of therapy (range, 2–10). All participants had prior treatment with a BTKi; 88% had undergone chemoimmunotherapy, and 85% had been double-exposed to a BTKi and a BCL2i. *TP53* aberrations were confirmed in 63% of participants, and 70% had unmutated IGHV (uIGHV) status. To date, data from only the expansion cohort have been presented. Epcoritamab therapy achieved an ORR of 61% and a CR rate of 39%, with a median response time of 2.0 months and a median time to CR of 5.6 months. No significant differences were observed among the subgroups of double-exposed patients, those with *TP53* aberrations, or those with uIGHV. The median PFS was 12.8 months, and the median OS had not been reached. The most common nonhematologic AEs included CRS (96%, with 17% grade 3; none led to therapy discontinuation), peripheral edema (48%), diarrhea (48%), fatigue (43%), and injection-site reactions (43%). Cytopenias were frequent, although most patients already had baseline anemia and thrombocytopenia. The four reported fatal AEs involved pneumonia, sepsis, and squamous cell carcinoma.

### 3.2. NVG-111

Another BsAb that has been extensively evaluated in recent years is NVG-111—a first-in-class, humanized, tandem scFv ROR1×CD3 bispecific T cell engager administered as a continuous intravenous infusion over 21 days, followed by 7 days off-drug. It is administered either in combination with ibrutinib or as monotherapy. In an open-label, dose-escalation study (#NCT04763083), 11 patients diagnosed with CLL—82% of whom had high-risk molecular features, including *TP53*, *ATM*, and *NOTCH1* mutations—and 3 patients with MCL received time-limited NVG-111 treatment. Among 12 patients evaluated for efficacy, 67% (n = 8) experienced an objective response, including 33% (n = 4) who achieved CR. Deeper responses were noted in patients who received additional cycles. Among all participants, the median PFS was 26.9 months. Among 13 patients evaluated for safety, adverse events occurred in all cases, most commonly during the first week. The most frequent were nausea (grade 1/2), headache, and fatigue. CRS of grade 1–2 was observed in 57% of patients, and ICANS (grade 2/3) in 14%. Grade 4 neutropenia was also observed in one patient [77].

### 3.3. Mosunetuzumab

The next promising molecule is mosunetuzumab—a CD20×CD3 BsAb approved by the FDA for treating R/R follicular lymphoma in adult patients who have undergone two or more lines of systemic therapy. There are three ongoing clinical trials studying mosunetuzumab as a potential treatment for patients with relapsed or refractory CLL (both as monotherapy and in combination with venetoclax), for patients with RT (as a first-line therapy with CHOP), and for CLL with MRD clearance (both as monotherapy and in combination with BTKi). None of the trial results have been published yet.

### 3.4. Glofitamab

Glofitamab is a CD20×CD3 BsAb with a unique 2:1 tumor-to-T-cell binding ratio, effectively redirecting a patient’s own T cells to destroy malignant B cells. Glofitamab therapy was found effective for DLBCL [78]. In the study by Carlo-Stella et al., 11 patients with RT, with a median age of 71 years, were treated with glofitamab as monotherapy. ORR and CR were 63.6% and 45.5%, respectively. CRS occurred in 72.7% of patients, mostly grade 1–2. ICANS-like neurologic AEs affected 5 patients, mostly grade 1. There were no fatal AEs or treatment discontinuations linked to glofitamab [79]. A Phase II study of glofitamab as monotherapy or in combination with polatuzumab vedotin, pirtobrutinib, or atezolizumab in RT is currently recruiting (#NCT06043674).

### 3.5. IGLV3-21^R110^-Directed Bispecific Antibody (R110-bsAB)

A clear characteristic of CLL is the expression of “stereotyped” B-cell receptors (BcRs) in a significant subset of patients. In approximately 30 to 35 percent of CLL cases, predominantly within the unmutated CLL subgroup, unrelated patients express a largely skewed immunoglobulin repertoire characterized by highly restricted and sometimes identical variable HCDR3 sequences [80]. The presence of identical BCRs among geographically distant patients strongly suggests that CLL ontogeny is actively driven by shared antigenic determinants, including specific autoantigens. Beyond shared structural features, stereotypy dictates similar antigen binding properties, functional responses, and distinct clinical outcomes [81,82,83]. Crucially, these stereotyped BCRs can autonomously signal in the absence of external antigens, and this independent function is vital for leukemia development and progression [84,85,86]. The continuous activation of downstream signaling cascades (such as BTK and PI3K) by these unique receptors provides the foundational biological rationale for utilizing targeted therapies, including specific monoclonal antibodies and kinase inhibitors, to disrupt leukemic cell survival [87,88]. IGLV3-21^R110^ is a specific genetic mutation in the BCR that generates a unique, tumor-specific neoepitope, promoting autonomous signaling in a high-risk, aggressive subset of CLL patients. While only 10 to 15 percent of all CLL patients express IGLV3-21^R110^, this marker is disproportionately common among patients needing therapy [89]. The therapeutic approach of R110-bsAb is to physically link this leukemia-specific neoepitope to the CD3 receptor on cytotoxic T cells, thereby inducing the formation of an immunological synapse and targeted cell destruction. R110-BsAb targeting the IGLV3-21^R110^ mutation and CD3 specifically eliminated both primary CLL cells and engineered cell lines overexpressing this neoepitope, using T cells from healthy donors and CLL patients as effectors. In vitro, R110-bsAb successfully maintained the viability of healthy polyclonal B cells and CD34+ hematopoietic stem cells. In vivo, R110-BsAb selectively removed CLL cells and cell lines expressing IGLV3-21^R110^, sparing normal peripheral blood mononuclear cells [90].

Many other agents are currently under investigation for CLL therapy, including GB261 (a CD20×CD3 BsAb), surovatamig/AZD0486 (a CD19×CD3 BsAb), and JNJ-75348780 (a CD3×CD22 BsAb).

**Table 2:** An overview of clinical trials assessing the safety and effectiveness of bispecific antibodies in CLL.

Agent & Target	Agent Characteristics	Clinical Trial Number	Trial Characteristics	No. of Participants
Epcoritamab	BsAb CD20×CD3 approved for DLBCL, FL and high-grade B-cell lymphoma	NCT04623541 (EPCORE CLL-1)	Phase I/II study on epcoritamab monotherapy in R/R CLL. ORR: 61%, CR: 39%, mPFS: 12.8 months, mOS: not reached [45].	23
			Phase I/II study on epcoritamab monotherapy in RT. ORR: 47.6%, CR: 40%, mPFS: 3.0 months, mOS: 13.0 months [75].	42
			Phase I/II study on epcoritamab with lenalidomide in RT. ORR: 82%, CR: 73%, mPFS: 5.7 months, mOS: not reached [76].	11
			Phase I/II study on epcoritamab with R-CHOP in RT ORR: 61%, CR: 39%, mPFS: 12.8 months, OS: not reached [76].	30
		NCT07108998	Phase II study on epcoritamab in CLL/SLL (consolidation therapy for 2nd generation BTKi ± obinutuzumab)	22 (estimated)
		NCT05791409	Phase I/II study on epcoritamab with venetoclax in R/R CLL/SLL. Estimated study completion in 2032.	112 (estimated)
		NCT06676033	Phase I study on epcoritamab in CLL and RT. Estimated study completion in 2027.	5
		NCT07218510 (LonGEVity Trial)	Phase II study on epcoritamab as a consolidation therapy for venetoclax and obinutuzumab in previously untreated CLL/SLL. Estimated study completion in 2029.	33 (estimated)
Mosunetuzumab	humanized BsAb CD3×CD20 approved for R/R FL	NCT05091424	Phase I study of mosunetuzumab alone or in combination with venetoclax in R/R CLL. Estimated study completion in 2030.	137 (estimated)
		NCT06926205	Phase II study of mosunetuzumab with CHOP as a first line in RT. Estimated study completion in 2028.	34 (estimated)
		NCT07052695	Phase I/II study of mosunetuzumab alone or in combination with BTKi in CLL/SLL. Estimated study completion in 2032.	40 (estimated)
		NCT02500407	Phase I/II study on mosunetuzumab monotherapy and with atezolizumab in R/RCLL and B-cell NHL. Trial completed.	713
GB261 CD20×CD3	BsAb CD20×CD3 computationally designed to maintain Fc effector function	NCT04923048	Phase I/II study in CLL and R/R B-cell NHL. Unknown status of completion.	460 (estimated)
AZD0486 (Surovatamig)	IgG4 fully human BsAb CD19×CD3	NCT06564038	Phase I/II study on AZD0486 as monotherapy or in combination with other anticancer agents in R/R CLL/SLL and other mature B-cell malignancies. Estimated study completion 2028.	276 (estimated)
NVG111 ROR1×CD3	first in class, humanized, tandem scFv, ROR1×CD3 BsAb	NCT04763083	Phase I study in R/R ROR1+ malignancies. Unknown status of completion.	90 (estimated)
ONO4685 PD1×CD3	First in class PD1×CD3 BsAb	NCT06547528	Phase I study in CLL/SLL and T-cell lymphoma. Estimated completion 2029.	108 (estimated)
JNJ 75348780 CD22×CD3	CD22×CD3 human BsAb	NCT04540796	Phase I study in R/R CLL NHL completed in 2025.	147

ORR—overall response rate; CR—complete remission; mPFS—median progression-free survival; mOS—median overall survival; CLL—chronic lymphocytic leukemia; RT—Richter transformation; SLL—small lymphocytic lymphoma; R/R—relapsed or refractory; BTKi—Bruton’s tyrosine kinase inhibitors; R-CHOP—rituximab, cyclophosphamide, doxorubicin, vincristine, prednisolone; NHL—Non-Hodgkin lymphoma; ROR1—receptor tyrosine kinase-like orphan receptor 1; CD—cluster of differentiation.

## 4. Cell Therapies

Over the past decade, CAR T-cell therapies have been successfully incorporated into treatment options for various hematologic malignancies, including B-cell non-Hodgkin lymphomas (NHLs) [91,92,93,94,95,96] and B-cell acute lymphoblastic leukemia [97,98]. The use of CAR T cells in CLL has been challenging. However, recent studies suggest they could be an effective treatment option for patients with relapsed or refractory disease (Table 3).

### 4.1. Challenges

The use of CAR T-cell therapy in CLL has encountered major challenges. While CAR T-cell therapy achieves long-lasting remissions in up to 60% of patients with acute lymphoblastic leukemia (ALL), the durable response rate is notably lower, at around 30%, in CLL. T cell exhaustion, an immunosuppressive TME and intrinsic T cell defects are key factors [35,36,99].

**Figure 2:**
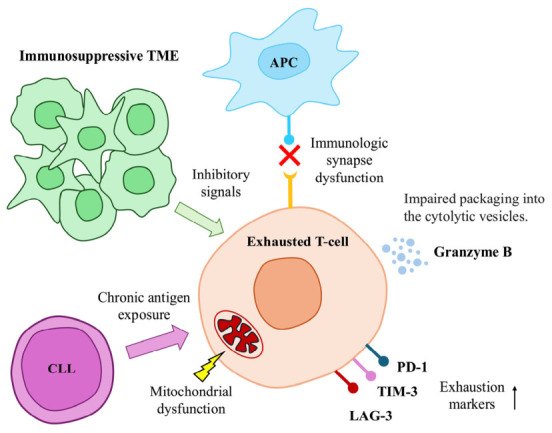
T-cell dysfunction in chronic lymphocytic leukemia. CLL—chronic lymphocytic leukemia; PD-1—programmed cell death protein 1; LAG-3—lymphocyte-activation gene 3; TIM-3—T-cell immunoglobulin and mucin containing protein-3; TME—tumor microenvironment; APC—antigen-presenting cell.

In CLL, T cells exhibit features of exhaustion (Figure 2). Riches et al. demonstrated that T cells from patients with CLL show increased levels of exhaustion markers, including CD244, CD160, and PD-1. Consequently, these T cells displayed impaired proliferation and cytotoxicity. This cytolytic deficiency is linked to defective granzyme loading into vesicles and disorganized, nonpolarized degranulation [35]. This T-cell dysfunction in CLL patients appears to be highly specific to CLL, as T cells targeting other antigens, such as CMV, maintain normal function and cytotoxicity [100]. Patients with CLL who show higher levels of exhaustion markers, including PD-1, TIM-3, and LAG-3, tend to have weaker responses to CAR T-cell therapy [101]. Present studies seek to find the underlying causes of CLL-induced T-cell exhaustion. The process is probably primarily driven by chronic, antigen-dependent cellular interactions within secondary lymphoid organs [38]. Within the lymph nodes, constant cellular interactions and exposure to interferon-gamma cause CLL cells and TME cells to upregulate PD-L1. Simultaneously, CD8^+^ T-cells progressively upregulate corresponding inhibitory receptors, such as PD-1, leading to a distinct accumulation of functionally exhausted PD-1^+^ CD8^+^ T cells in the lymph nodes rather than in the PB [38,102]. Alongside the PD-1/PD-L1 axis, T-cell dysfunction is mediated by sialic acid-binding Ig-like lectin 10 (Siglec-10) ligands CD24 and CD52 on CLL cells, as well as Galectin-9 secretion by CLL cells and some myeloid cells [39,103]. Galectin-9 binds to the TIM-3 inhibitory receptor present on exhausted T cells. This Galectin-9/TIM-3 interaction further compounds the inability of T cells to control the CLL by promoting the development of suppressive CD4^+^ regulatory T-cells, ultimately facilitating the tumor’s escape from immune surveillance [103]. Additionally, CLL-associated monocytes exacerbate this suppressive environment by secreting inflammatory and immunosuppressive cytokines such as IL-10, TNF-α, and CXCL9. Crucially, in vivo depletion of these myeloid cells not only controls CLL progression and reduces systemic inflammation, but also successfully repairs the exhausted T-cell phenotype, restoring normal immune cell function [102]. Indoleamine 2,3-dioxygenase-secreting myeloid-derived suppressor cells, which are increased in CLL, are also known to inhibit T cell activity in vitro and promote the development of regulatory T cells [104].

The TME supports the growth and survival of CLL leukemic cells. It offers a supportive niche, and its interactions with CLL cells promote disease progression and reduce treatment effectiveness. The TME includes stromal cells that sustain cell-to-cell communication and aid leukemia cell growth, as well as immune-suppressive cells such as nurse-like cells, myeloid-derived suppressor cells, and regulatory T cells [105]. Nurse-like cells interact with CLL cells, decreasing their SDF-1 receptors and thus preventing spontaneous apoptosis [106]. Resistance mechanisms contribute to CLL refractoriness. Therefore, new CAR T-cell therapies should be designed to overcome them. Efforts to use CAR T-cell therapy in CLL include combining therapeutic agents, optimizing CAR design, and employing allogenic CAR T-cells.

Gene expression analyses have highlighted biological differences between CAR T cells from CLL patients who achieve CR and those who do not. In responders, the infused CAR T cells exhibit a distinct memory-like genetic profile and marked activation of the IL-6 and STAT3 signaling pathways. Conversely, T cells from non-responders are dominated by gene signatures associated with terminal effector differentiation, altered metabolism, exhaustion, and apoptosis. The capacity for sustained remission is linked to the baseline presence of memory-like CD27^+^CD45RO^−^CD8^+^ T cells prior to CAR T manufacturing. Effective CAR T cells actively secrete STAT3-associated cytokines, with serum IL-6 levels directly correlating with robust in vivo expansion. Disruption of the IL-6/STAT3 pathway significantly impairs CAR T-cell function and proliferation. Ultimately, a specific subset of functional CAR T cells (CD27^+^PD-1^−^CD8^+^) expressing high levels of the IL-6 receptor appears to drive tumor eradication and serves as a strong predictor of clinical success [101]. CD8^+^ T cell dysfunction in CLL extends to the metabolic level. Chronic exposure to the leukemic microenvironment leads to impaired glucose uptake via reduced GLUT1 reserves and altered mitochondrial homeostasis. Upon stimulation, these T cells fail to undergo proper mitochondrial biogenesis due to decreased PGC-1α levels. The clinical relevance of this metabolic exhaustion is profound: studies evaluating pre-infusion CD19-directed CAR T-cells have shown that increased mitochondrial mass is a hallmark of complete responders. The mitochondrial fitness of these cells directly correlates with their ability to expand and persist in vivo. Therefore, enhancing mitochondrial biogenesis during CAR T processing might rescue T-cell functionality and improve therapeutic outcomes in CLL [107]. Direct cell-to-cell contact with CLL cells severely impairs actin polymerization in both CD4^+^ and CD8^+^ T cells. This structural defect prevents the proper recruitment of key regulatory proteins, ultimately crippling the formation of a functional immunological synapse. This contact-dependent mechanism can induce synapse dysfunction even in healthy allogeneic T-cells [108].

To overcome the severe in vivo T-cell exhaustion in CLL, modern CAR T-cell engineering extends beyond basic antigen receptor expression. The selection of an appropriate intracellular costimulatory domain is critical for therapeutic success. Clinical observations in CLL indicate that incorporating 4-1BB costimulatory signaling into CAR T cells yields superior therapeutic outcomes compared with CD28 costimulatory signaling [109,110,111]. Unlike CD28, 4-1BB signaling actively promotes mitochondrial biogenesis, enhances oxidative phosphorylation, and protects the engineered cells from rapid exhaustion, thereby ensuring the long-term in vivo persistence required for durable remissions [109,112]. To actively combat the immunosuppressive TME and reduce systemic toxicity, current research focuses on developing fourth-generation “armored” CAR T cells (often referred to as TRUCKs, T cells redirected for universal cytokine-mediated killing), which are engineered to co-express potentially enhancing cytokines [113,114]. Therapy with CAR T cells carries the risk of severe, life-threatening immune-mediated toxicities, such as CRS and ICANS. One strategy to prevent serious adverse outcomes is to equip the transferred cells with a specialized safety, or ‘suicide’ gene. One of the approaches being studied is the inducible caspase 9 (iCasp9) safety switch system. By co-expressing this suicide gene, clinicians can give a specific small-molecule dimerizing agent to quickly trigger targeted apoptosis of the CAR T cells in vivo, providing an essential, on-demand way to neutralize the therapeutic product and safely manage uncontrollable toxicities [115,116].

### 4.2. Autologous CD19 CAR T Cells

#### 4.2.1. Lisocabtagene Maraleucel

**Figure 3:**
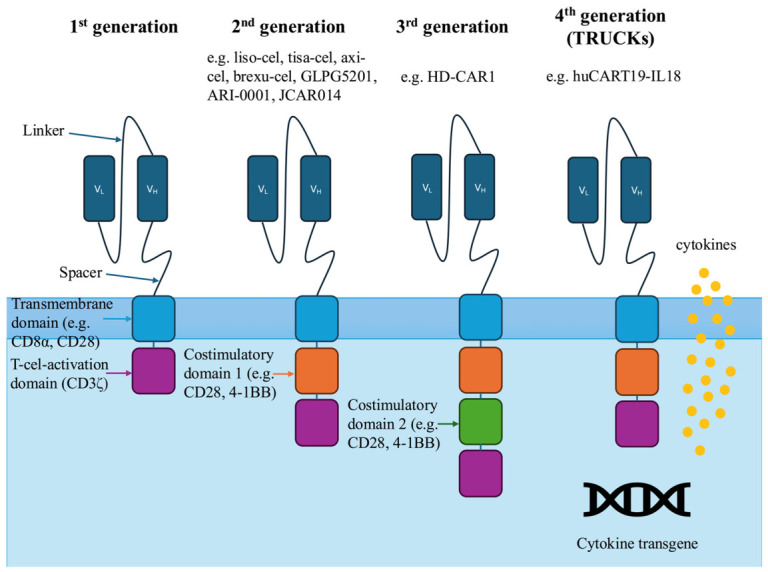
Chimeric antigen receptor structure and different generations of CAR T cells. First-generation CAR T cells—featuring a single intracellular T-cell-activation domain. Second-generation CAR T cells—featuring a single costimulatory domain alongside the primary signaling domain. Third-generation CAR T cells—featuring two distinct costimulatory domains alongside the primary signaling domain. Fourth-generation CAR T cells—engineered for transgenic cytokine release. V_L_—variable light chain; V_H_—variable heavy chain; TRUCKs—T cells redirected for universal cytokine-mediated killing.

Lisocabtagene maraleucel (liso-cel) is an autologous CD19-targeted CAR T-cell therapy composed of defined and equal doses of CD8^+^ and CD4^+^ CAR-positive T cells with a 4-1BB costimulatory domain. Lisocabtagene maraleucel (liso-cel) is an autologous CD19-targeted CAR T-cell therapy made up of equal and precise doses of CD8^+^ and CD4^+^ CAR-positive T cells with a 4-1BB costimulatory domain (Figure 3) [117]. In March 2024, the FDA approved liso-cel for patients who have progressed after at least two lines of therapy, including a BTKi and BCL-2 inhibitor. The approval was based on promising data from the TRANSCEND CLL 004 trial (phase I/II) [44]. One hundred and seventeen CLL patients with BTKi failure, including 70 with venetoclax failure, received liso-cel. Patients were treated with two dose levels of liso-cel: 50 × 10^6^ CAR T cells or 100 × 10^6^ CAR T cells. In the efficacy-evaluable group (n = 96), the ORR was 48%, with 18% of patients achieving a CR or CRi. Among those who progressed on a BTKi and did not respond to venetoclax, response rates were similar—an ORR of 43% and a CR rate of 18%. The efficacy-evaluable population had a median DOR of approximately 35 months, and the median PFS was 11.9 months. For patients previously treated with both a BTKi and venetoclax, the median DOR was also 35 months, while the median PFS was 12 months. In double-refractory patients, uMRD was achieved in 64% of cases in PB and 59% in BM (at 10^−5^ sensitivity). Ten patients (9%) experienced grade 3 CRS; none had grade 4 or 5 CRS. Twenty-one patients (18%) experienced grade 3 neurotoxicity, and one patient (1%) experienced grade 4 neurotoxicity. At 24-month follow-up, disease responses remained durable, with consistently high rates of uMRD and no new safety concerns [118].

#### 4.2.2. Tisagenlecleucel

Tisagenlecleucel (tisa-cel), a second-generation anti-CD19 CAR T-cell therapy featuring a 4-1BB costimulatory domain and CD8α hinge, was the first CD19-targeting CAR T-cell therapy to demonstrate early success in B-cell malignancies [110]. In the trial, 14 patients with R/R CLL received tisa-cel. At a median follow-up of 19 months, the ORR was 57%, with 4 CRs and 4 PRs. Among the 14 treated and evaluable patients, median OS was 29 months, with an 18-month OS rate reaching 71%. The median PFS was 7 months, and the 18-month PFS was 28.6%. The infusions were generally well tolerated. Neutropenia-related complications, such as fever, and delayed CRS were the most common treatment-related AEs. A recent follow-up confirmed that two patients remain in MRD-negative remission 10 years after treatment, with detectable circulating CAR T cells [119].

#### 4.2.3. Axicabtagene Ciloleucel

Axicabtagene ciloleucel (axi-cel) is another autologous anti-CD19 CAR T-cell therapy. It also utilizes the CD28 domain but does not involve 4-1BB, unlike liso-cel and tisa-cel. Cappell et al. reported long-term results of axi-cel therapy in seven heavily pretreated CLL patients. They observed an ORR of 88% and a CR rate of 63%. The median duration of sustained response was 82 months, with 50% of the patients maintaining a response for more than three years [120].

#### 4.2.4. Brexucabtagene Autoleucel

Brexucabtagene autoleucel (brexu-cel) has the same structure as axi-cel; however, it undergoes an additional T cell selection process during manufacturing to remove leukemic cell contamination [94]. In the ZUMA-8 trial, 15 enrolled patients were divided into four cohorts: cohorts 1 and 2 received brexu-cel at doses of 1 × 10^6^ and 2 × 10^6^ cells/kg, respectively; cohort 3 included patients with low tumor burden; and cohort 4 included patients receiving ibrutinib as their last therapy. With a median follow-up of 24.3 months, one dose-limiting toxicity (grade 4 CRS) was reported in cohort 3. Grade ≥ 3 neurologic events occurred in 3 patients (20%). The ORR reached 47%, including one CR (7%). All cohort 3 patients responded. CAR T-cell expansion occurred in 4 patients (27%). CAR T-cell expansion and responses were observed in patients with low tumor burden. Due to suboptimal CAR T-cell expansion in most patients, the ZUMA-8 trial was terminated early [121].

#### 4.2.5. GLPG5201

GLPG5201 is a second-generation CAR T-cell therapy targeting CD19, featuring a 4-1BB costimulatory domain, administered as a one-time fixed intravenous dose. It was evaluated in EUPLAGIA-1—a Phase I/II, open-label, multicenter trial involving patients with R/R CLL, R/R SLL, and RT. In December 2024, the initial safety and efficacy results were presented. All 15 enrolled patients had R/R CLL, and 9 also had RT. Of the 15 efficacy-evaluable patients, 13 responded (86.7% ORR), and 10 achieved CR (66.7%). At a median follow-up of 6 months, 8 of the 10 patients who achieved CR (80%) maintained ongoing CR. GLPG5201 demonstrated a favorable safety profile, with most grade ≥ 3 treatment-related AEs being hematological. No grade ≥ 3 CRS or any-grade ICANS was observed [122].

#### 4.2.6. huCART19-IL18

huCART19-IL18, an armored CAR T-cell therapy engineered to target CD19 and secrete interleukin-18 to boost antitumor effects, was tested in 21 patients with R/R NHLs. The safety profile was manageable: CRS occurred in 62% of patients, mostly mild or moderate (47% grade 1–2), and ICANS was reported in 14%, all grade 1–2. Importantly, no unexpected AEs were observed. CAR T-cell expansion was strong across all dose levels. At 3 months after infusion, ORR was 81%, with 52% achieving a CR. After a median follow-up of 17.5 months, the median DOR was 9.6 months [113].

#### 4.2.7. Varnimcabtagene Autoleucel

Varnimcabtagene autoleucel (ARI-0001) is an autologous second-generation CAR T-cell therapy targeting CD19 with 4-1BB costimulation, developed entirely at Hospital Clínic in Barcelona, Spain. It was approved in Spain for treating relapsed/refractory B-cell ALL. From 2017 to 2024, 13 patients with relapsed/refractory CLL and 16 with RT received ARI-0001. CRS occurred in 90% of patients; however, only 3.4% experienced grade ≥ 3 cases. Just 2 cases (6.9%) of ICANS were reported, all grade ≤ 2. In the CLL group, the ORR and CR rates were both 84.6%. With a median follow-up of 1.3 years, the median DOR, PFS, and OS were not reached. The median duration of circulating CAR T cells was 3 years [123].

#### 4.2.8. JCAR014

JCAR014, another CD19 CAR T-cell therapy with a 1:1 ratio of CD8^+^ to CD4^+^, was tested in a phase I/II clinical trial, which enrolled 47 R/R CLL patients, including 9 with prior or current RT. It was found that 94% of patients had high-risk cytogenetics. Median follow-up was 79.6 months. Median DOR was 18.9 months. Median PFS was 8.9 months, and the 6-year PFS was 17.8%. By day 28, OR and CR rates were 70% and 17%, respectively. The 6-year DOR and OS were 26% and 31%, respectively. CRS and neurotoxicity occurred in 82% (14% grade ≥ 3) and 33% (27% grade ≥ 3) of patients, respectively [124].

#### 4.2.9. HD-CAR-1

HD-CAR-1 is a third-generation autologous CD19-directed CAR T-cell product that includes two costimulatory domains, CD28 and 4-1BB. It was tested in a phase I/II trial involving patients with double R/R CLL. By day 90, six patients (67%) achieved a CR, with five (83%) achieving uMRD. With a median follow-up of 27 months, 2-year PFS and OS were 30% and 69%, respectively. In non-responders, there was a significant increase in effector memory-like CD8^+^ T cells with high expression of CD39 and/or CD197. Treatment-related toxicity was remarkably low, with only one case of grade 3 CRS and no neurotoxicity [125].

#### 4.2.10. BAFF-R CAR T Cell Therapy

Another potential target is BAFF-R, a specific marker involved in B-lymphocyte development and the survival of mature B cells [126]. BAFF-R is especially significant in CLL because it is highly expressed in the clonally expanded population of mature B cells [127]. BAFF-R CAR T-cell therapy showed cytotoxic activity against both CLL cell lines and primary B-cells from CLL patients. Additionally, these CAR T cells were effective against CD19-knockout CLL cells that resist CD19 CAR T-cell therapy [128]. Two phase I clinical trials are currently recruiting patients to evaluate BAFF-R CAR T-cell therapy for the treatment of R/R CLL (#NCT06191887, #NCT06916767).

### 4.3. Dual-Target CAR T Cells

The development of dual-targeting methods marks a significant step forward in preventing antigen escape and improving therapeutic outcomes. CD19/CD22-targeting CAR T cells were tested in two patients who developed DLBCL-RT two years after CLL diagnosis. Both patients remained in CR (17 and 7 months post-infusion), showing good tolerability and no severe infections. Both experienced grade 1 CRS. Patient 1 exhibited rapid CAR T-cell expansion, while patient 2 showed moderate expansion [129]. A Phase I/II study of CD19/CD22-targeting CAR T cells for treating R/R CD19/CD22-positive B-ALL and various B-cell lymphomas is currently recruiting (#NCT06834529).

Safety and outcomes of lentiviral bispecific anti-CD20/anti-CD19 (LV20.19) CAR T cells were assessed in 14 patients with R/R RT and CLL. All experienced CRS, and 93% required tocilizumab. Sixty-four percent of patients developed immune effector cell-associated hemophagocytic lymphohistiocytosis-like syndrome (IEC-HS), a hyperinflammatory complication of immune effector cell-based therapies characterized by macrophage activation features such as cytopenias, hyperferritinemia, and coagulopathy. Among these, two patients with CLL experienced grade 3 and grade 4 IEC-HS. At day 28, the ORR was 92% among evaluable patients (n = 13). All responders (n = 12) had disease-negative BM. With a median follow-up of 11 months, only one RT patient relapsed. The median DOR was not reached, and the median OS was 15 months. Although LV20.19 CAR T cells showed efficacy in CLL and RT, the high incidence of IEC-HS in CLL patients raises concerns [130].

Other dual-targeting CAR T-cell therapies are currently being studied, including CD19/BAFF-R dual-targeted CAR T cells [131].

### 4.4. Triple-Target CAR T Cell Therapy

Targeting three B-cell antigens simultaneously represents a new strategy to prevent antigen-negative relapse. The safety of administering CAR T cells that target CD19, CD20, and CD22 (TriCAR19.20.22 T cells) [132] will be assessed in patients with R/R CLL, with or without RT. CAR T cells will be infused following an immunosuppressive conditioning regimen consisting of fludarabine and cyclophosphamide (#NCT07166419).

### 4.5. Allogeneic CAR T Cells

Allogeneic CAR T cells can act as an “off-the-shelf” alternative to autologous CAR T-cell therapy. Instead of being derived from a patient’s own T cells, they are prepared in advance from healthy donor cells. This approach allows for immediate treatment, which is crucial for patients with rapidly progressing leukemia, and eliminates the risk of leukemic cell contamination that can happen when using a patient’s own cells [133].

CARCIK-CD19 is a donor-derived, CD19-targeted, cytokine-induced CAR T-cell therapy engineered using the non-viral Sleeping Beauty transposon system [134]. A Phase I/II clinical trial evaluating its safety and clinical activity in patients with CLL is currently recruiting (#NCT05869279). The ARDENT trial is evaluating SC291, an “off-the-shelf”, hypoimmune-modified, CD19-directed allogeneic CAR T therapy for R/R B-cell malignancies, including CLL. The CAR T cells feature HLA depletion and CD47 overexpression to prevent rejection (#NCT05878184). Another investigational allogeneic CAR T therapy for CLL is ALLO-501A. It involves disrupting the TCRα constant gene to reduce the risk of graft-versus-host disease (GvHD) and editing the *CD52* gene, thereby enabling the use of a humanized anti-CD52 monoclonal antibody to selectively deplete host T cells while sparing donor CAR T cells [135]. The safety and efficacy of ALLO-501A in patients with R/R CLL are being assessed in the ALPHA-2 trial (#NCT04416984).

CTX112 is an allogeneic, CRISPR-Cas9-engineered CAR T-cell therapy that involves five precise edits: inserting an anti-CD19 CAR into the TRAC locus and disrupting the *TRAC*, *B2M*, *TGFBR2*, and *ZC3H12A* genes to prevent GvHD, evade immune rejection, resist immunosuppression, and improve persistence [136]. The Phase I/II open-label, multicenter trial is assessing the safety and efficacy of CTX112 in patients with relapsed or refractory (R/R) B-cell malignancies, including CLL.

### 4.6. Combination Therapies with CAR T Cells

#### 4.6.1. Ibrutinib

Combining CAR T-cell therapy with ibrutinib is gaining attention for its potential to enhance efficacy and minimize adverse effects. Ibrutinib improves the survival of activated T cells, lowers Treg/CD4^+^ ratios, and inhibits CLL-mediated immunosuppression through both BTK-inhibitor-related and unrelated mechanisms [137]. Simultaneous administration of ibrutinib improves both engraftment and therapeutic efficacy of anti-CD19 CAR T cells in murine models of CLL [138]. Ibrutinib also decreases CRS caused by CAR T cells [139]. In a study by Gauthier et al., 19 CLL patients received ibrutinib ≥2 weeks before leukapheresis and continued it for at least 3 months post-CAR T-cell therapy. At 4 weeks, the iwCLL 2018 ORR was 83%, with 61% achieving MRD-negative marrow by IGH sequencing. Concurrently, ibrutinib reduced CRS severity and cytokine levels compared to CAR T-only treatment [140]. Liso-cel combined with ibrutinib for R/R CLL/SLL was also assessed in the dose-escalation cohort of the phase I/II TRANSCEND CLL 004 study. Among 19 patients with ≥1-month follow-up, 95% responded. All responses were received by day 30, and 89% remained ongoing for ≥6 months. Eighty-nine percent of patients achieved MRD negativity in PB samples by flow cytometry, and 79% did so in BM by NGS. No dose-limiting toxicities occurred. Ibrutinib-related AEs were observed in 79% of patients, with 37% ≥ grade 3. AEs led to dose reductions in 2 patients and discontinuations in 4. CRS occurred in 74% of patients (1 grade 3), and 32% experienced neurological events (3 ≥ grade 3). Tocilizumab and/or corticosteroids were required for management in 42% of patients [141]. Gill et al. conducted a single-center phase II trial adding autologous huCART-19 cells to ibrutinib in CLL patients who did not achieve CR after ≥6 months of ibrutinib. Primary endpoints were safety, feasibility, and CR within 3 months. Of 20 enrolled patients, 19 received huCART-19. Median follow-up was 41 months. CRS occurred in 18 patients (grades 1–2 in 15, grade 3 in 2, grade 4 in 1), and 5 experienced neurotoxicity (grades 1–2 in 4, grade 4 in 1). The 3-month CR rate was 44%, and the 12-month MRD negativity was 72%. The estimated 48-month OS and PFS were 84% and 70%, respectively. Of 15 patients with early MRD negativity, 13 remained in ongoing CR. In CLL patients who did not achieve CR with ibrutinib alone, huCART-19 induced deep and durable remissions [142].

#### 4.6.2. Lenalidomide

An immunomodulatory agent, lenalidomide, is also being studied in combination therapies. When used with CD23-targeted CAR T cells, lenalidomide maintained CAR T-cell cytotoxicity, cytokine production, proliferation, and the formation of immune synapses between CAR T and CLL cells in vitro. In a xenograft model of CLL, this combination improved CAR T-cell migration to leukemic sites and slowed disease progression [143].

#### 4.6.3. PI3K Inhibition

PI3K inhibitors can cause T cell-mediated autoimmunity, and it is hypothesized that temporarily inhibiting PI3K during CAR T-cell production could improve their effectiveness in patients with CLL. The impact of adding the dual PI3Kδ/γ inhibitor duvelisib during CAR T-cell manufacturing was evaluated. Duvelisib-treated CAR T therapy (Duv-CAR-T) normalized CD4/CD8 ratios; increased the number of T-stem cell memory, naïve, and memory cells; and boosted cytotoxicity against CD19^+^ CLL. In CLL xenografts, Duv-CAR-T cells expanded more, eliminated leukemia more rapidly, persisted longer, and improved survival compared with conventional CAR T cells [144].

### 4.7. Allogeneic CAR NK Cells

CAR NK cells are increasingly recognized as a possible alternative to CAR T cells. CAR NK cells offer a significantly improved safety profile; their distinct cytokine dynamics and shorter lifespan inherently reduce the risk of severe CRS and long-term toxicities. They possess a unique dual-targeting advantage: they can eradicate leukemic cells through both the engineered CAR construct and their native innate receptors via ADCC, thereby mitigating the risk of tumor escape due to antigen downregulation. Because NK cells can mediate a potent graft-versus-leukemia (GvL) effect without inducing GvHD, they can be safely utilized in an allogeneic setting. This allows for their generation from diverse healthy sources, including PB, umbilical cord blood, and NK cell lines, ultimately providing an “off-the-shelf” therapeutic product that bypasses the fundamental limitations of patient-specific CAR T manufacturing [145,146,147].

Liu et al. demonstrated that CD19-targeted CAR NK cells effectively eliminated CD19-positive leukemia cells in vitro and significantly extended survival in a xenograft mouse model [148]. In the dose-escalation phase of the phase I/II trial, the anti-CD19 CAR NK cells derived from cord blood were administered to 11 patients with R/R NHL or R/R CLL. None of the patients developed CRS, neurotoxicity or GVHD. Of the 11 patients treated, 73% responded to therapy, with 64% achieving CR [149]. The expansion phase included 26 additional patients. In total, 37 patients were treated. No significant toxicities, including CRS, neurotoxicity, and GvHD, were reported. The ORR at days 30 and 100 was 49% each, while the 1-year OS and PFS rates were 68% and 32%, respectively. Patients who achieved a response showed greater CAR NK-cell expansion and longer persistence [150].

**Table 3:** Clinical trials assessing specific cell therapies for CLL patients.

Agent	Phase	Target	No. of Participants	ORR	CR/CRi	PFS Rate	OS Rate	Safety	Ref.
Lisocabtagene maraleucel (liso-cel)	I/II	CD19	117	47%	18%	Median: 11.9 months	Median 30.3 months	CRS Gr. ≥ 3: 9%; NT Gr. ≥ 3: 19%	[44]
Tisagenlecleucel (tisa-cel)	I	CD19	14	57%	29%	Median: 7 months	Median: 29 months	Neutropenia fever, delayed CRS	[110]
Axicabtagene ciloleucel (axi-cel)	I/II	CD19	7	88%	63%	N/A	Median OS not reached	N/A	[120]
Brexucabtagene autoleucel (brexu-cel)	I	CD19	15	47%	13%	N/A	N/A	CRS Gr. 4: 7%; NT Gr. ≥ 3: 20%	[121]
GLPG5201	I/II	CD19	15	93%	66.7%	N/A	N/A	No grade ≥ 3 CRS or any-grade ICANS were observed	[122]
huCART19-IL18	I	CD19	21 (R/R NHL)	81% at 3 months	52%	N/A	N/A	CRS: 62% (Gr. ≥ 3: 15%); ICANS: 14% (all Gr. 1–2)	[113]
Varnimcabtagene autoleucel (ARI-0001)	I	CD19	13 (CLL cohort)	84.6%	84.6%	60% at 4 years	Not reached	CRS: 90% (Gr. ≥ 3: 3.4%); ICANS gr. 2: 6.9%	[123]
JCAR014	I/II	CD19	47	70%	17%	Median: 8.9 months	Median: 25 months	CRS: 82% (Gr. ≥ 3: 14%); NT: 33% (Gr. ≥ 3: 27%)	[124]
HD-CAR-1	I/II	CD19	9	67%	67%	30% at 2 years	69% at 2 years	CRS gr. 3: 9%; no NT	[125]
LV20.19	I	CD20/CD19	14 (RT and CLL)	92%	46%	N/A	Median: 15 months	CRS: 100% (Gr. ≥ 3: 14.3%); ICANS: 21% (gr. 3: 14.3%); IEC-HS: 64% (Gr. ≥ 3: 14.3%)	[130]
Liso-cel + Ibrutinib	I	CD19	19	95%	63%	N/A	N/A	CRS: 74% (Gr. ≥ 3: 5%); NT: 32% (Gr. ≥ 3: 16%)	[141]
huCART-19 + Ibrutinib	II	CD19	19	83%	43% at 3 months	84% at 4 years	70% at 4 years	CRS: 94.7% (Gr. ≥ 3: 15.8%); NT: 26.3% (Gr. 4: 5.3%)	[142]
Anti-CD19 CAR-NK	I/II	CD19	37	49%	37.8%	32% at 1 year	68% at 1 year	No significant CRS, NT, or GvHD	[150]

ORR—overall response rate; CR—complete remission; CRi—complete remission with incomplete count recovery; N/A—not available; PFS—progression-free survival; OS—overall survival; Ref.—reference; Gr.—grade; RT—Richter transformation; CLL—chronic lymphocytic leukemia; CRS—cytokine release syndrome; NT—neurotoxicity; ICANS—immune effector cell-associated neurotoxicity syndrome; IEC—immune effector cell–associated hemophagocytic lymphohistiocytosis-like syndrome; GvHD—Graft-versus-Host Disease.

## 5. Conclusions

The therapeutic approach to CLL has undergone a major shift, moving from chemotherapy to chemoimmunotherapy and now to highly effective, mechanism-based targeted agents. The widespread use of BTKis and BCL2is has significantly decreased dependence on traditional anti-CD20 monoclonal antibodies, which are now mainly used in specific clinical situations. However, monoclonal antibodies remain an important biological platform, and the discovery of new targets like ROR1, BAFF-R, and other disease-specific antigens highlights their ongoing importance. Because of their generally favorable safety profiles, some of these novel targeted antibodies have a high likelihood of entering the general armamentarium for standard CLL care. Next-generation antibodies may even change how we combine treatments, especially in overcoming resistance and improving immune-based tumor control.

More importantly, the future of CLL therapy is increasingly likely to be shaped by immunotherapeutic methods that actively engage cellular cytotoxicity. Bispecific antibodies capable of redirecting native T cells have shown the ability to induce deep responses regardless of traditional resistance pathways in CLL and RT patients, making them a highly promising and scalable strategy. Similarly, CAR T-cell therapies offer the possibility of profound and potentially long-lasting disease eradication, challenging the traditional idea of continuous treatment in CLL. However, their clinical application must be carefully weighed against real-world patient demographics. Because CLL primarily concerns older, frail patients who are unlikely to withstand highly aggressive treatments like CAR T-cell therapy, these intensive cellular approaches will likely be reserved for a minority of relatively young or fit patients presenting with aggressive or refractory disease. Advances in CAR design, toxicity reduction, and manufacturing platforms are expected to expand their use and enhance therapeutic effectiveness, potentially making them a ready-to-use, “off-the-shelf” option as well.

In parallel, NK cell-based therapies are emerging as a promising complementary approach. NK cells with innate, MHC-independent antitumor activity may offer a better safety profile, creating opportunities for “off-the-shelf” cellular treatments and innovative combination strategies. The integration of NK-cell platforms with antibody engineering and cellular redirection technologies could be a key next step in advancing immunotherapy.

Despite their transformative potential, many important questions remain unanswered. The best way to sequence and combine BsAb, CAR T cells, and NK-cell therapies compared to established targeted drugs is still unknown, especially regarding long-term effectiveness and safety. Identifying which patients benefit most, balancing benefits against immune-related side effects, and determining if these treatments can lead to long-lasting remission without ongoing therapy requires careful prospective studies. Future advances will rely on biomarker-based trial designs, a better understanding of immune escape mechanisms, and strategic combination approaches. In summary, although small-molecule inhibitors currently form the foundation of CLL treatment, the future clearly points toward cellular immunotherapies. Bispecific antibodies, CAR T cells, and NK-cell strategies are set not only to support existing treatments but also to potentially transform therapeutic goals. Finding their best use now is one of the most important priorities in today’s CLL research.

## Data Availability

No new data were created or analyzed in this study. Data sharing is not applicable to this article.
